# Residual force enhancement in humans: Is there a true non‐responder?

**DOI:** 10.14814/phy2.14944

**Published:** 2021-08-02

**Authors:** Florian K. Paternoster, Denis Holzer, Anna Arlt, Ansgar Schwirtz, Wolfgang Seiberl

**Affiliations:** ^1^ Department of Sport and Health Sciences Biomechanics in Sports Technical University of Munich Munich Germany; ^2^ Department of Human Sciences Human Movement Science Bundeswehr University Munich Neubiberg Germany

**Keywords:** eccentric muscle action, electrical stimulation, force enhancement, history‐dependence, lengthening contraction, voluntary muscle action

## Abstract

When an active muscle is stretched and kept isometrically active, the resulting force is enhanced compared to a purely isometric reference contraction at the same muscle length and activity; a generally accepted muscle property called residual force enhancement (rFE). Interestingly, studies on voluntary muscle action regularly identify a significant number of participants not showing rFE. Therefore, the aim was to unmask possible confounders for this non‐responsive behavior. Ten participants performed maximum voluntary isometric plantarflexion contractions with and without preceding stretch. Contractions were accompanied by the assessment of voluntary activation using the twitch‐interpolation technique. The same test protocol was repeated four additional times with a least on day rest in‐between. Additionally, at the first and fifth sessions, a submaximal tetanic muscle‐stimulation condition was added. At both muscle‐stimulation sessions mean rFE higher 10% (*p* < 0.028) was found. In contrast, during voluntary muscle action, individual participants showed inconsistent rFE across sessions and only one session (#3) had significant rFE (5%; *p* = 0.023) in group means. As all participants clearly had rFE in electrical stimulation conditions, structural deficits cannot explain the missing rFE in voluntary muscle action. However, we also did not find variability in voluntary activation levels or muscle activity as the confounding characteristics of “non‐responders.”

## Graphical abstract

Four familiarization sessions are not sufficient to constantly show residual force enhancement in novice subjects. This seems to be mainly affected by voluntary control of eccentric and post‐eccentric muscle action. From a muscle structural point of view, all participants proofed to have the ability to produce residual force enhancement, when the muscles are artificially activated using tibial nerve stimulation.

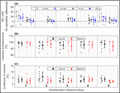

## 1 INTRODUCTION

The phenomenon of residual force enhancement (rFE) was first described in 1952 in a study by Abbott and Aubert (1952). The authors tested the toad sartorius muscle with varying contraction conditions. They identified the interesting phenomenon that after an active lengthening with the muscle kept active isometrically, the steady‐state force after this lengthening was higher than compared to a pure isometric contraction at the same muscle length and activation level. Although the exact mechanism(s) of this stretch‐induced force enhancement (FE) are not fully understood, the phenomenon itself is thought to be a generic part of human muscle function. This is supported by experimental evidence across all structural levels from muscle fiber to in vivo multi‐joint leg extensions (Edman et al., 1982; Hahn et al., 2010; Paternoster et al., 2016; Pinnell et al., 2019; Rassier et al., 2003; Seiberl et al., 2015). In current literature, a key mechanism of stretch‐induced rFE is attributed to the giant molecular spring Titin, which is assumed to become stiffer and shorter during an active eccentric contraction, thereby increasing passive on top to active force production (Herzog, 2018; Rode et al., 2009).

The more studies on stretch‐induced history‐dependent effects involved voluntary human muscle action, the more unexpected “non‐responder” behavior was reported for some of the participants (see review: 2). This means that after voluntary eccentric muscle action not all participants showed enhanced forces or torques in steady‐state phases after lengthening compared to isometric references. For example, in 2005 Oskouei and Herzog reported, that only eight out of 17 participants showed rFE following a submaximal voluntary lengthening contraction (Oskouei & Herzog, 2005), and more examples can be found in the literature (Hahn et al., 2007; Oskouei & Herzog, 2006a; Seiberl et al., 2012; Tilp et al., 2009). In this context, the influence of contraction intensity on this phenomenon remains a matter of debate. There is literature showing a decreasing number of non‐responders with increasing contraction intensity of adductor pollicis muscle (Oskouei & Herzog, 2005), whereas other studies do not see changes in the number of non‐responders when changing contraction intensities in knee‐extensors (Seiberl et al., 2012) or dorsiflexors (Paquin & Power, 2018). Thus, there might be an influence of the investigated muscle or muscle group and complexity of muscle control. It is also worth noting that the relative but not the absolute amount of rFE seems to be independent of the contraction intensity (Oskouei & Herzog, 2006b; Paquin & Power, 2018; Seiberl et al., 2012). Some further interesting insights were given by two studies published regarding the influence of a training intervention on rFE (Chen & Power, 2019; Hinks et al., 2021). Chen and Power (2019) compared the influence of a 4‐week concentric versus eccentric muscle training intervention on the development of rFE. Interestingly, mean rFE only increased in the concentric training condition. This was explained by a reduction in the number of non‐responders (four subjects) in the concentric group and an increased co‐activation of antagonistic muscles in the eccentric group. Hinks et al. (2021) did an isomeric training intervention and found inconsistent results regarding non‐responders. Some non‐responders turned into responders until the end of the training intervention and vice versa.

Nevertheless, considering the knowledge extracted from in vitro, in situ and stimulated human muscle experiments, rFE is seen as a very robust property of skeletal muscle (Edman et al., 1978; Hisey et al., 2009; Leonard et al., 2010; Rassier et al., 2003) and characterized as follows: rFE increases with increasing stretch amplitude, is independent of stretch velocity and occurs over the entire range of the force–length relationship, thus even can exceed the isometric force at optimal muscle length (Edman et al., 1978; Julian & Morgan, 1979; Peterson et al., 2004; Rassier et al., 2003). Of course, in vitro and in situ experiments do have very special experimental conditions. Fibers are usually enclosed in a highly concentrated calcium solution ensuring optimal contraction conditions (Tomalka et al., 2021). The in situ activation of the muscle is performed using electrical stimulation thereby avoiding motor unit recruiting based on Henneman's size principle (Henneman et al., 1965). Another important fact is that during experiments with muscle preparations or fibers, active lengthening of the contractile unit (a prerequisite for rFE) can easily be controlled and standardized. This is way more difficult in in vivo muscle‐tendon unit contractions and flexion on the joint level not necessarily always lead to stretch of the muscle (Aeles & Vanwanseele, 2019). Thus, it is not surprising that in vivo experiments on voluntary muscle action show a less clear picture in their observations, and—as mentioned above—even identify participants that do not respond to active lengthening with increased force at all (Power et al., 2020; Seiberl et al., 2015). Seiberl et al. speculated that either “[…] non‐responders lack certain muscle physiological abilities to generate enhanced force or […] they are limited in performance during and after lengthening as a result of neural inhibitions or insufficient task specific motor control” (Seiberl et al., 2015). In this context, neuronal inhibition is related to a reduced descending drive or incomplete motor unit recruitment and firing rates (Babault et al., 2001; Beltman et al., 2004; Gandevia, 2001).

This study was designed to test parts of these speculations. We were specifically interested in the question, if non‐responding participant's muscles do not have the abilities to show rFE (during artificial activation), or if subjects merely are unable to voluntarily control eccentric and post‐eccentric contractions sufficiently. We hypothesized that if the latter was the case and there are no muscle structural issues for non‐responders, artificially activating muscles in those non‐responders should result in clear rFE. Furthermore, in literature, the possibility of neural inhibition that hiders full force potential during eccentric muscle action is commonly discussed (Babault et al., 2001; Beltman et al., 2004). Consequently, such an inhibition during active lengthening might also reduce the torque output in the post‐eccentric steady‐state phase by influencing motor unit recruitment or discharge patterns. To see whether the voluntary activation level is reduced after an active stretch, the interpolated twitch technique was used (Merton, 1954). Additionally, we were interested in if becoming a responder can be trained by task familiarization. In this study, we compared electrically versus voluntarily activated eccentric and post‐eccentric (rFE conditions) muscle action of physically active participants, that were not specifically familiarized with the stretch‐contraction task. And we repeated the testing on four additional days to see if there are changes in stretch responses of participants, due to increasing task familiarization.

## 2 MATERIALS AND METHODS

### 2.1 Participants

In total, 11 physically active participants (25 ± 2 years|87 ± 23 kg|183 ± 13 cm|two females) with no acute ankle joint injuries or neurological disorders took part in the study. The study was conducted according to the declaration of Helsinki. The study was a side‐project of a long‐term research project which has Ethics approval, sharing the same methods (Holzer et al., 2020). Therefore, no separate approval was obtained. Participants participated voluntarily and gave written informed consent.

### 2.2 Experimental setup

**FIGURE 1:**
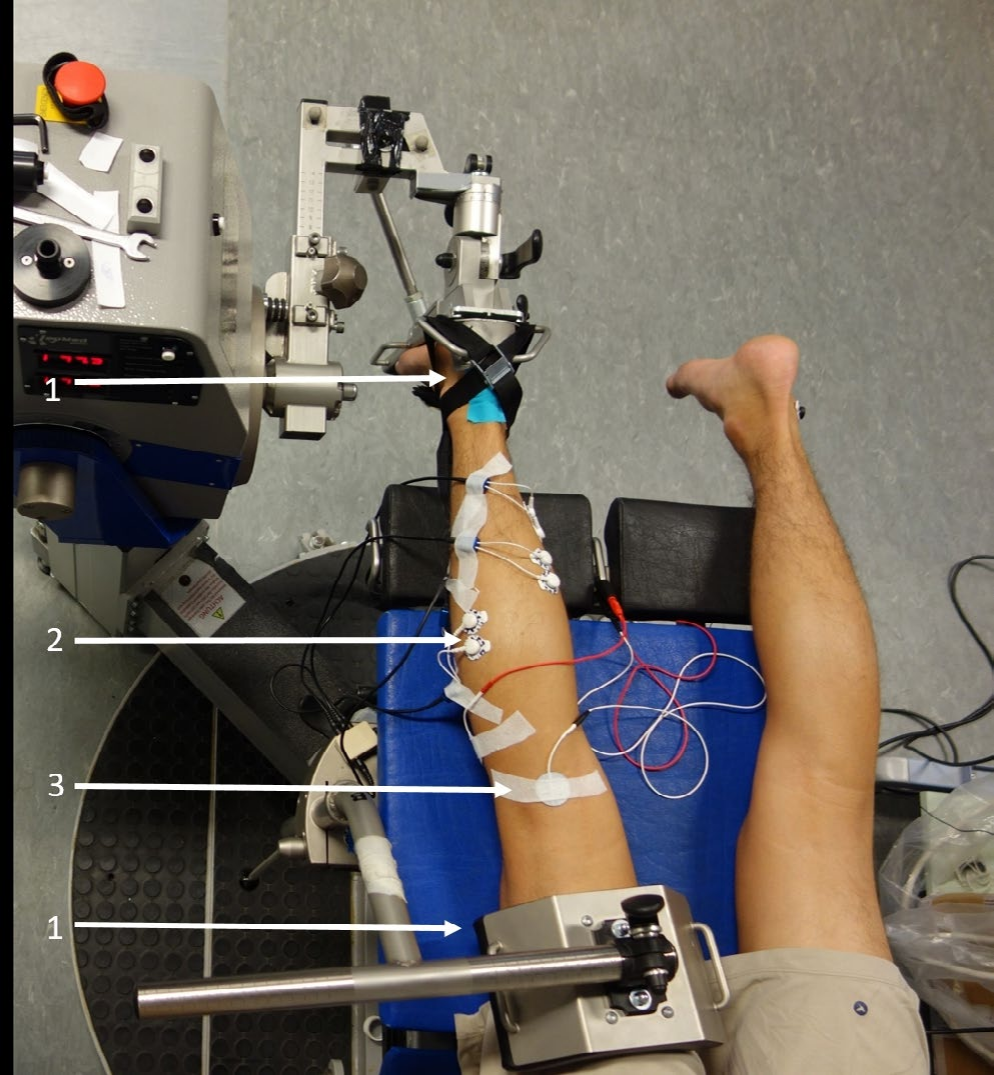
Experimental setup: (1) Fixation of foot and thigh. (2) Electromyography. (3) Electrical stimulation

A motor‐driven dynamometer (IsoMED 200; D&R Ferstl) was used to measure plantar flexion torque at 1000 Hz. Participants lay prone on the bench of the dynamometer with the knee and hip fully extended. The right foot was fixed toward the footrest with an inelastic flat band to avoid heel displacement (Figure 1). To minimize unwanted movement of the participants during contractions adjustable table straps and pads were used for shoulder, hip, and thigh (Figure 1). The rotational axis of the dynamometer was aligned with the ankle joint axis. The muscle activity of the tibialis anterior (TA), soleus (SOL), gastrocnemius lateralis, and medialis (GL, GM) was recorded with a wired electromyography (EMG) system at 1000 Hz (EMG USB2; OT Bioelectronica). The reference electrode was placed on the lateral malleolus of the contralateral foot. Skin preparation and electrode placement were performed according to the SENIAM guidelines (Hermens et al., 1999).

### 2.3 Electrical stimulation

**FIGURE 2:**
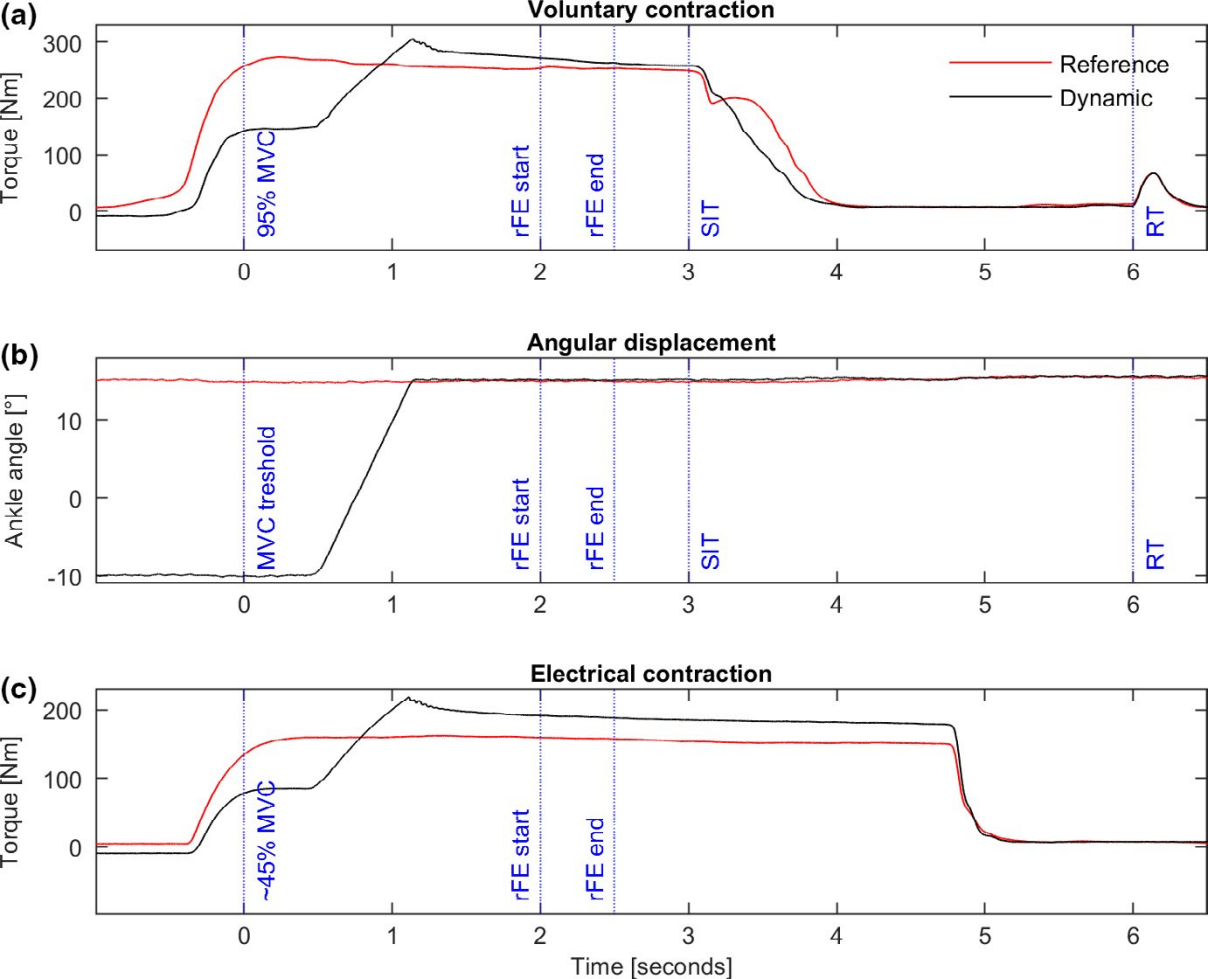
Overview data analysis. Exemplar data of voluntary contraction (a), angular displacement (b) and electrical contraction (c). 95%/45% MVC = Individual time‐point when participant reached 95/45% of maximum voluntary contraction torque level. Dynamic (black), isometric‐eccentric‐isometric contraction; Reference (red), pure isometric contraction; RFE start/end, analysis window to calculate mean torque to get residual force enhancement; RT, time‐point of supra‐maximal stimulation at rest; SIT, time‐point of supra‐maximal stimulation during contraction

A constant current stimulator (DS7AH; Digitimer) was used for the electrical stimulation of the nervus tibialis in the popliteal fossa (cathode). The anode was attached to the fibula head of the ipsilateral leg. The electrical stimulus intensity for the maximum twitch‐response was assessed during a protocol with increasing stimulus current. The final intensity, where twitch‐response torque did not further increase, was multiplied by 1.5 to evoke supramaximal stimulus intensity for the following experiment. During the voluntary contraction conditions, participants received two supramaximal twitches (doublets, 10 ms apart): The first twitch was released during contraction, 3 s after the participants reached 95% of their individual maximum torque (superimposed twitch response; SIT). After the first twitch, participants were instructed to fully relax their muscles and another 3 s after SIT, the second twitch happened to assess the resting twitch (RT; Figure 2). Voluntary activation was then calculated as $\mathrm{VA}\left(\%\right)=\left(1‐\frac{\mathrm{SIT}}{\mathrm{RT}}\right)*100$ (Merton, 1954).

In addition to the estimation of VA, tetanic muscle stimulation (frequency: 50 Hz; pulse width: 1000 μs; duration: 5 s) was used in the first and last sessions to test the structural properties of the muscles. The intensity of tetanic stimulation was increased until a steady torque level between 40% and 50% of individual maximum isometric voluntary contractions (MVC) torque was reached in the reference position of 15° dorsiflexion (0° refers to tibia axis perpendicular to the plantar aspect of the foot).

### 2.4 Experimental protocol

Prior to the first test session, participants had a training session to get familiar with the test situation, dynamometer, and test procedures. Clear instructions on how the plantar flexion strength tests need to be performed were given in order to gain a high reproducibility of torque output during MVC at different angular positions.

The training session was followed by five identical test sessions. In the test sessions, if necessary, participants were visually informed about their individual torque traces and improvable aspects were addressed (e.g., no muscle deactivation during the active stretch phase). This feedback was used to increase the familiarization effect to the isometric–eccentric–isometric muscle action. Each session started with an unspecific warm‐up consisting of 5 min of cycling on an ergometer. After the general warm‐up, a specific warm‐up of the plantar flexors was performed on the dynamometer that also served for preconditioning of the muscle‐tendon unit (Maganaris, 2003) in order to increase comparability between trials and days. Thereafter, the sessions started with 2–3 MVC contractions (~3 s, 3 min break) at 15° dorsiflexion and 10° plantarflexion. Participants were asked to push as hard and fast as they can. The reached a torque level served for the settings of dynamometer control and electrical stimulation in the following experiment.

The experimental test protocol then consisted of three maximum voluntary isometric–eccentric–isometric contractions (dynamic condition) and three isometric contractions without a preceding stretch (15° dorsiflexion; reference condition to dynamic stretch contractions ending at 15° dorsiflexion), that were performed in a randomized order. The trials were separated by a rest period of 3 min to prevent fatigue (Salles et al., 2009). The dynamic contractions started at an angular position of 10° plantarflexion, followed by an active stretch of 25° at 40°*s^−1^ to reach the reference position. The angular displacement during the dynamic contractions started 0.5 s after participants rising torque output passed a value of 95% of previously assessed MVCs at the respective joint angle. Control of dynamometer displacement as well as timing of electrical stimulation during voluntary trials was done using a self‐written MATLAB script. In the dynamic and reference condition, contractions lasted ~4 s (Figure 2).

In sessions one and five, the test protocol additionally incorporated two submaximal tetanic muscle stimulation trials with identical settings as for the MVC dynamic and reference condition. The five different sessions were separated by at least 1 day of rest. For all MVC contractions, the examiner used identical verbal instructions and gave maximum verbal encouragement.

### 2.5 Data analysis

Data were captured using the Nexus software (Vicon) and transferred to MATLAB (The Math Works, Inc.) for further analysis.

All torque data were smoothed using a 50 ms moving average. For the calculation of FE during stretch, the peak torque value of the active stretch phase of the dynamic condition (ecc_peak) and the reference condition were used (iso_peak). FE was calculated as $\left(\frac{\mathrm{ecc}\_\mathrm{peak}}{\mathrm{iso}\_\mathrm{peak}}‐1\right)*100$. The calculation of rFE, enhanced torque in the steady‐state after lengthening, was performed using $\left(\frac{\mathrm{ecc}\_\mathrm{steady}}{\mathrm{iso}\_\mathrm{steady}}‐1\right)*100$, where ecc_steady and iso_steady represent the mean steady‐state torque between 2 and 2.5 s after the participants passed the increasing torque level of 95% MVC (Figure 2) in the dynamic and isometric reference condition, respectively (Figure 2). For FE and rFE, positive values indicate the percentage of enhanced torque during or after stretch, compared to the isometric condition.

To estimate the voluntary activation level, SIT and RT were calculated as follows: SIT was calculated as the peak torque value within 0.5 s after the stimulation minus the baseline torque level (mean of 50 ms time window) just prior to the stimulus. RT was calculated in the same way just with a 3‐s delay after SIT, when participant's muscles were fully relaxed. As mentioned earlier, stimulations were released automatically using a self‐written MATLAB script.

EMG data were offset corrected, filtered (Bandpass: 20–499 Hz, fourth order), rectified, and smoothed with a moving average of 250 ms. The time‐points used for the statistical analysis of the muscular activity in dynamic and reference trials were the same as for the calculation of rFE. All EMG data were normalized using the peak of the smoothed EMG signal from the best MVC contraction in the reference position.

The coefficient of variation (CV) was calculated using peak torque values of the dynamic and reference condition of all three trials per session. CV was calculated as $\frac{\mathrm{SD}}{\mathrm{mean}}*100$ and is expressed as a percentage value.

### 2.6 Statistics

For further statistical analysis of the parameters FE and rFE (and related muscle activity), only the data of the best trial per condition were used. The best trail was defined by the trial with highest torque during stretch for the dynamic conditions and the trial with the highest torque during isometric steady‐state for the reference contractions.

Values above or below two standard deviations were treated as outliers and excluded from the statistical analysis. The normality of data was checked using the Shapiro–Wilk test. To analyze the results within a session (for electrical stimulation also between sessions one and five), paired sample *t*‐tests were used including Cohen's *d* effect size referencing 0.2 as small, 0.5 as medium, and >0.8 as large effect (Cohen, 1988). The development of FE, rFE, voluntary activation level, and CV for the voluntary contractions across sessions was analyzed using a one‐way repeated measures ANOVA (five levels = different sessions). If sphericity was violated, Greenhouse–Geisser correction was used. The effect size is presented as *ω* ^2^. The correlation between FE and rFE was performed using Spearman's rank correlation. A *p* ≤ 0.05 indicates a significant difference. The JASP software was used for statistical analysis (JASP Team, 2019). Data are presented as mean ± SD.

## 3 RESULTS

Results (mean ± SD) are based on 10 participants, as there was one dropout, which did not finish the study. We removed this subject from the entire analysis. For some results (statistics of EMG), detailed analysis can be found in the Supporting Information file.

### 3.1 Electrical stimulation

**FIGURE 3:**
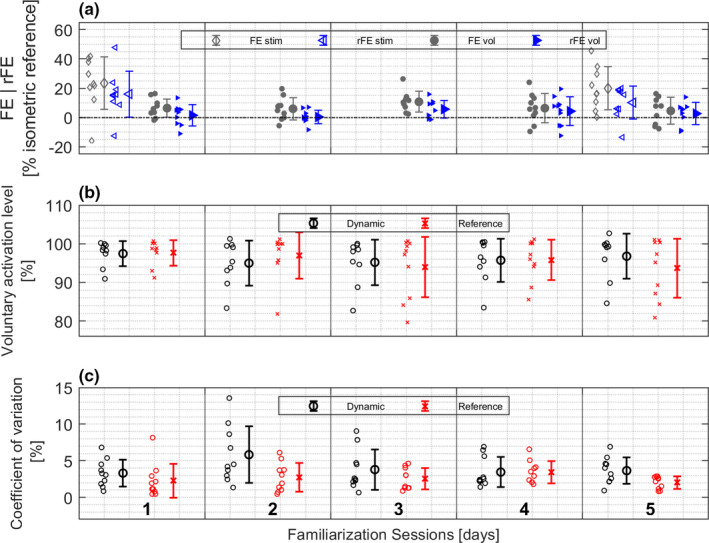
Mean, SD and individual results for the five familiarization sessions. (a) residual Force enhancement (rFE) and Force enhancement (FE) for stimulated (stim) and voluntary (vol) contractions. (b) Voluntary activation level. (c) Analysis of the coefficient of variation of the peak torque values. For (b) and (c) “Dynamic” represents isometric‐eccentric‐isometric contractions and “Reference” represents pure isometric contractions

At the end of the active stretch (FE) as well as in the isometric steady‐state phase (rFE), significantly increased torque values (Table 1) compared to isometric reference were found in the first (FE: 23.4 ± 17.9%. rFE: 16.0 ± 15.7%) as well as in the fifth session (FE: 20.0 ± 14.7%. rFE: 10.2 ± 11.1%). There was no difference in the amount of FE and rFE between sessions one and five (FE: *t*(7) = −0.19, *p* = 0.86, *d* = −0.07. rFE: *t*(7) = 0.49, *p* = 0.642, *d* = 0.17; Figure 3a).

**TABLE 1:** Mean ± SD of submaximal electrical stimulated plantar flexion torque (~40%–50% MVC) at two different sessions on two different days. Related statistical output indicates significant differences between isometric–eccentric–isometric (dynamic) and purely isometric (reference) contractions

	Peak torque (Nm)				Steady‐state torque (Nm)		
	During Stretch	Isometric Reference	Statistics	After Stretch		Isometric Reference	Statistics
Session 1	193.0 ± 56.9	156.3 ± 39.4	*t*(8) = 3.57, *p* = 0.007, *d* = 1.19	161.4 ± 45.2		141.5 ± 42.8	*t*(8) = 4.18, *p* = 0.003, *d* = 1.39
Session 5	198.4 ± 74.6	163.4 ± 47.6	*t*(8) = 3.13, *p* = 0.014, *d* = 1.04	170.1 ± 59.0		154 ± 51.0	*t*(8) = 2.69, *p* = 0.028, *d* = 0.90

*p* ≤ 0.05 indicates a significant difference. *d* = Effect size after Cohen (Cohen, 1988).

Abbreviation: MVC, maximum isometric voluntary contractions.

### 3.2 Voluntary contraction

Significant voluntary FE at the end of stretch was present in the first (6.4 ± 6.1%), second (6.0 ± 7.6%), and third session (10.7 ± 7.1%). For the forth (6.4 ± 9.9%) and fifth (4.5 ± 9.1%) session, comparable but insignificant amounts of FE were found (Table 2). Significant voluntary rFE (Table 2) was only measurable in session three, resulting in an rFE value of 5.6 ± 6.0%. For the other session, no enhanced torque value was observed (1: 1.4 ± 7.4%. 2: 0.3 ± 4.5%. 4: 4.3 ± 9.8%. 5: 2.6 ± 7.6%; Table 2). There was no change regarding the amount of FE and rFE in between the sessions (FE: *F*(4, 28) = 0.77, *p* = 0.553, *ω* ^2^ = 0.00. rFE: *F*(4, 24) = 0.45, *p* = 0.774, *ω* ^2^ = 0.04; Figure 3a).

**TABLE 2:** Mean ± SD of maximum voluntary plantar flexion torque at five different sessions on 5 days with at least 1 day rest in between. Related statistical output indicates significant differences between isometric–eccentric–isometric (dynamic) and purely isometric (reference) contractions

	Peak torque (Nm)		Statistics	Steady‐state torque (Nm)		Statistics
	During stretch	Isometric reference		After stretch	Isometric reference	
Session 1	326.0 ± 95.8	305.7 ± 82.5	*t*(9) = 2.86, *p* = 0.019, *d* = 0.90	302.7 ± 100.1	295.5 ± 80.4	*t*(8) = 0.84, *p* = 0.423, *d* = 0.28
Session 2	327.7 ± 100.2	309.7 ± 92.9	*t*(9) = 2.27, *p* = 0.049, *d* = 0.72	295.1 ± 96.6	294.2 ± 93.6	*t*(9) = 0.21, *p* = 0.839, *d* = 0.07
Session 3	347.0 ± 98.3	314.8 ± 94.8	*t*(8) = 4.90, *p* = 0.001, 1.64	309.8 ± 96.8	295.7 ± 101.1	*t*(8) = 2.80, *p* = 0.023, *d* = 0.93
Session 4	347.6 ± 103.6	328.4 ± 99.8	*t*(9) = 2.18, *p* = 0.057, *d* = 0.69	322.6 ± 108.5	309.0 ± 96.5	*t*(9) = 1.51, *p* = 0.166, *d* = 0.48
Session 5	338.1 ± 107.6	324.2 ± 100.8	*t*(8) = 1.52, *p* = 0.166, *d* = 0.51	314.1 ± 99.3	307.2 ± 98.2	*t*(8) = 0.96, *p* = 0.363, *d* = 0.32

*p* ≤ 0.05 indicates a significant difference. *d* = Effect size after Cohen (Cohen, 1988).

The voluntary activation level was not different between the dynamic and reference condition in each of the five sessions (*p* > 0.118; Figure 3b) ranging between 97.5% and 95.0% for the dynamic task and between 97.7 and 94.0% for the reference condition.

Regarding muscle activity in the steady‐state phase, GM activity in session 1 was lower for the dynamic compared with the reference condition (6.2 ± 6.8%, *t*(7) = −2.60, *p* = 0.035, *d* = −0.92), whereas this was not the case for the other sessions (*p* > 0.221). GL showed a lower activity for session two (14.3 ± 16.6%, *t*(8) = −2.58, *p* = 0.032, *d* = −0.86) and session three (7.0 ± 9.0%, *t*(9) = −2.77, *p* = 0.022, *d* = −0.88) in the dynamic compared with the reference condition. The other sessions showed no significant differences for GL (*p* > 0.098). Like for GL, SOL showed a significantly reduced activity in the dynamic conditions for session two (9.2 ± 8.1%, *t*(8) = −3.44, *p* = 0.009, *d* = −1.15) and session three (6.3 ± 8.3%, *t*(9) = −2.38, *p* = 0.041, *d* = −0.75). Sessions one, four, and five showed no differences between conditions (*p* > 0.108). For TA, there was a reduced activity for the dynamic condition in session three compared to the reference condition (7.8 ± 8.5%, *t*(8) = −2.76, *p* = 0.025, *d* = −0.92). No difference in TA activity was found in all other sessions (*p* > 0.086). For more detailed EMG analysis see Table S1.

In sessions two and five, the CV of peak torques showed significant differences comparing the dynamic with the isometric condition (Session 2: *t*(9) = 3.02, *p* = 0.015, *d* = 0.95, Session 5: *t*(9) = 2.42, *p* = 0.033, *d* = 0.80; Figure 3c). In all other sessions, no differences in CV between conditions were found (*p* > 0.229). In addition CV did not change over time and hence was comparable in all sessions (Reference: *F*(4, 36) = 1.35, *p* = 0.269, *ω* ^2^ = 0.02. Dynamic: *F*(4, 36) = 2.04, *p* = 0.109, *ω* ^2^ = 0.06).

**FIGURE 4:**
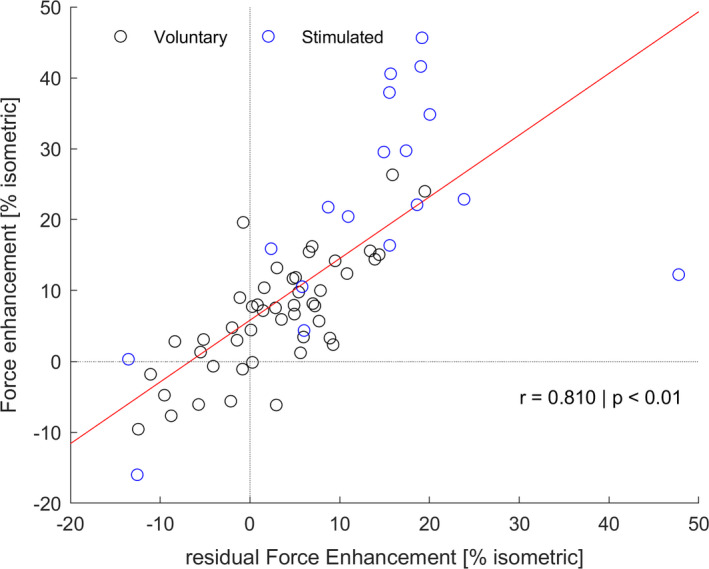
Correlation of residual Force enhancement and Force enhancement. Analysis incorporates the data of voluntary and electrically stimulated trials of all sessions

The analysis of all FE and rFE cases (including electrical and voluntary trails) revealed a strong correlation (*p* < 0.01, *r* = 0.81) between the FE at the end of the stretch and the rFE after stretches (Figure 4).

## 4 DISCUSSION

The aim of this study was to clarify whether the absence of rFE in some participants really can be attributed to individual physiological muscle abilities or merely is to be attributed to volitional motor control. To answer the question, we used three approaches. First, possible lack of certain muscle physiological abilities was analyzed using tetanic stimulation to eliminate the influence of voluntary motor control. Second, the comparison between artificial and voluntary muscle contraction helped to distinguish between pure muscle function and neuromuscular abilities. Third, by testing participants with no test‐specific experiences on five different days, we wanted to evaluate if task‐specific familiarization can explain the missing of rFE.

### 4.1 Artificial muscle contraction

For electrical stimulation, results showed clearly enhanced torque values during active stretch (session one: 23.4%, session five: 20.0%), as well as rFE of more than 10% in the isometric steady‐state phase after active lengthening (Table 1). In addition, the amount of rFE and FE did not differ comparing session one and session five (Figure 3). This indicates that there was no structural adaptation in terms of a training effect between the first and the last session, although this was very unlikely to occur within such a short period of time anyway. Compared to previous literature, the amount of rFE of the electrically stimulated plantar flexors is in line with previously published studies ranging between 9 and 15% (Fukutani et al., 2017, 2019; Pinniger & Cresswell, 2007).

Most importantly, these results on electrically stimulated muscle action clearly confirm that each and every participant's plantar flexor had the ability to produce stretch‐induced torque enhancement. This was not surprising and the findings on FE and rFE during electrical stimulation were expected. Comparable results can be found in many studies on human muscle function (Cook & McDonagh, 1995; Ruiter et al., 2000). Our results, therefore, further affirm the assumption that rFE is a fundamental property of human muscle function. So far the favored explanation in the recent literature is the engagement of passive structures within the sarcomere thereby focusing on the spring‐like molecule titin (Herzog et al., 2016; Rode et al., 2009; Tomalka et al., 2020). However, the exact underlying mechanism(s) are still not fully understood.

### 4.2 Voluntary contractions

In contrast to the stimulated contractions, results regarding FE and rFE were less consistent for the voluntary muscle contractions. In fact, true rFE (considered as significantly enhanced group mean torque) was only evident in one session (session three, ~5% rFE, Table 2). This phenomenon of participants not showing enhanced torque during and after lengthening has previously been reported among many studies (Hahn et al., 2007; Oskouei & Herzog, 2005, 2006a; Seiberl et al., 2012; Tilp et al., 2009). As shown above, we observed clear FE and rFE in electrically stimulated trials of all and a lack of certain muscle physiological abilities cannot be assumed to play a role in the absence of rFE in these participants. For the voluntary plantarflexion contractions, literature shows rFE of ~9% (Dalton et al., 2018; Hahn et al., 2012; Pinniger & Cresswell, 2007). This value is bigger than in our study (~5% session 3), despite having a comparable range of motion (sensitivity of rFE toward stretch amplitude; Hisey et al., 2009) and similar activation level (Dalton et al., 2018; Hahn et al., 2012).

### 4.3 Neuromuscular aspects

It is known from the previous work that the amount of generated rFE after the stretch is related to the amount of increased force/torque during active stretch (Bullimore et al., 2007; Paternoster et al., 2016). This is once more supported by the strong correlation between FE and rFE in this study (Figure 4). Except for one data point, the upper‐right quadrant of Figure 4 clearly shows that whenever a participant showed rFE, there always was FE in preceding stretch. This indicates that if we want to understand the variability of rFE in voluntary muscle action, a key aspect is the eccentric stretch phase and product stretch‐induced FE.

Generally, the absence of increased eccentric forces or torques compared to isometric references is a well‐known problem in voluntary muscle action. In a recent review, Hahn (Hahn, 2018) argued that FE is possible in voluntary human muscle but several factors have to be considered. Two possible explanations are mostly discussed: First, neural inhibitory effects due to eccentric loading of the muscle. And second, activation dynamics, that is, the time it takes to fully activate the muscle. Concerning the latter, activation dynamics influence the generation of high eccentric forces. A muscle simply needs time to fully activate and generate force, which can take 300–500 ms (Bobbert & Ingen Schenau, 1990). If muscle activation is starting at the same time as the eccentric lengthening starts, the muscle is in submaximal states during the lengthening and the resulting force cannot be maximal (Hahn, 2018). This can be overcome by pre‐activating the muscle before stretch. This has the additional advantage that fascicles have more time to take up tendon compliance during the pre‐activation, thereby minimizing initial shortening (Raiteri & Hahn,), to ensure a real fascicle stretch during the eccentric phase (Hahn, 2018). This is an important point to consider because it is known that rFE is sensitive to the amount of stretch (Abbott & Aubert, 1952; Cook & McDonagh, 1995; Edman et al.,; Hisey et al., 2009). The importance of the pre‐activation phase and rFE was recently shown by Fukutani et al. (2019). To account for these confounding effects, we used a pre‐activation protocol in our study, where a trigger preload of 95% MVC was used in voluntary contractions to ensure that all eccentric trials started with controlled pre‐activation of the muscle. Activation dynamics, therefore, can be excluded as a confounder in our study that would explain the absence of FE and rFE in voluntary contractions.

Thus, especially the inhibitory effects during eccentric muscle action need to be discussed as confounding neuromuscular factors. It is reported that eccentric loading can result in a reduced descending drive or incomplete motor unit recruitment and firing rates (Gandevia, 2001). It might be speculated that inhibitory effects on neural muscle activation influence our results on volitional muscle force production during stretch with an impact on the isomeric steady‐state phase after stretch. Using the twitch‐interpolation technique, we could not find any differences in voluntary activation between dynamic and reference conditions in the five sessions (Figure 2). Therefore, the participants' individual capacity to voluntarily activate their muscles in the steady‐state phase stayed constant and cannot explain the missing rFE. However, it needs to be discussed if the ITT method used on whole triceps muscle‐tendon unit is sensitive enough to identify possible inhibition at a peripheral level. Analysis of muscle activity in individual parts of the triceps, for example, showed that in session two, SOL and GL had a lower muscle activity during the dynamic task compared to the isometric reference contraction. This can be interpreted as partial inhibition. Important to note here is that the same amount of torque as for the isometric condition was produced. Thus, after active stretch, the same amount of torque was produced with less muscle activation. This “activation reduction” also represents a well‐accepted history depended effect related to the mechanisms of rFE and was previously reported in various publications (Jones et al., 2016; Paternoster et al., 2017; Seiberl et al., 2016). In addition, the lower EMG value of GM might also underestimate the amount of rFE in session one and session three. However, since there were also sessions (four and five) where no differences in the muscle activity of triceps parts were present (partial), inhibitory effects cannot solely explain the absence of rFE in voluntary muscle action (at least on the level of surface EMG analysis). In a 4‐week training intervention study by Chen and Power (2019), it was shown that neuronal aspects might play a role with respect to the non‐responder phenomenon. Thereby, concentric training erased all non‐responders, whereas this was not the case within the eccentric training group. Especially at the beginning of an intervention, gains in muscle strength are preliminary associated with neuronal adaptions (Moritani & deVries, 1979). Therefore, specific neuronal capabilities might play a role in the non‐responder phenomenon; even so, this cannot explain the inter‐day variability of some subjects in the current study. Instead, no clear picture could be drawn concerning the effect of isometric training on the development of rFE (Hinks et al., 2021). During their 8‐week lasting isometric training, Hinks et al. (2021) found some responders at the beginning of the intervention that turned into non‐responders at the end, some non‐responders that turned into responders, and some participants that did not change their status. These scattered results are similar to our findings, showing a day‐to‐day variability in terms of being a responder for some subjects.

### 4.4 Task‐specific abilities

Besides neuromuscular factors, Seiberl et al. (2015) also proposed the missing of task‐specific control to explain this discrepancy between stimulated versus voluntary contractions, both maybe related to a participant's familiarization with the isokinetic test.

For a generalized interpretation of our results concerning possible positive familiarization effects, we focused on statistical group means. However, the repeated measures ANOVA did not find differences in voluntary FE and rFE levels across test days. As we barely found enhanced torques in isometric–eccentric–isometric conditions, a familiarization effect of the (at first) unexperienced participants toward performance enhancements in voluntary eccentric muscle action cannot be attested within our time period of five consecutive sessions (Figure 3a). Besides neuromuscular factors, as proposed above, task‐specific abilities might play an important role. Poor task‐specific motor control without familiarization would be evident in high fluctuations of peak torque values across different days as well as in different conditions (isometric vs. eccentric). Accordingly, low within‐ and between‐day fluctuations would indicate a good and stable neuro‐mechanical task‐specific control of the participants. Analysis of the CV of peak torque values showed no differences across time for both conditions. Hence CV was not influenced by the five familiarization sessions. For the individual sessions, the second and last sessions showed a higher CV for the dynamic task (Figure 3c) compared with the reference condition. In both cases, the CV of the dynamic task showed higher values. However, with a maximum CV of 5.8% for the dynamic and 3.4% for the isometric task values good reliability of torque production can be assumed (Roth et al., 2017). This indicates at least stable task‐specific skills of the participants with respect to peak torque values, and cannot explain the variability of rFE throughout various sessions.

### 4.5 Limitations

A limitation of the study is the missing of a direct measurement of the fascicle length change during the eccentric phase. An active stretch of the contractile element is essential in the context of rFE to trigger suggested mechanisms such as an increase in titin stiffness (Fukutani & Herzog, 2019). However, taking current literature into account (Bakenecker et al., 2020; Chapman et al., 2021; Fukutani et al., 2019), it seems unlikely that there was no fascicle lengthening, as pre‐activation before stretch in these studies, as also used in our study, led to clear fascicle elongation during the active muscle‐tendon unit lengthening. Additionally, Bakenecker et al. (2020) found that greater preloads increase the amount of fascicle stretch. As we used preloads of 95% MVC for voluntary stretch contractions, we think that the rotation of the dynamometer induced a lengthening of the fascicles to thus fulfill the prerequisite of rFE.

## 5 CONCLUSION

This is the first study testing the influence of task familiarization on rFE. The aim was to gain insights into the phenomenon of rFE responders and non‐responders. As a result, we showed that for subjects, that are well trained but not experienced with isokinetic devices, four familiarization sessions are not sufficient to constantly show rFE (for individual data see Figure S1). Using artificial muscle activation, we found rFE for all subjects. Hence, from a muscle structural point of view, all participants proofed to have the ability to produce rFE, when the muscles are artificially activated using tibial nerve stimulation. It seems that the absence or variability in showing rFE in voluntary contractions is not directly explainable by task‐specific abilities like the reproduction of peak torque values during different conditions or neural inhibition during eccentric muscle loading. Hence, despite being known for so many years, rFE still keeps secrets, especially for voluntary muscle action.

## CONFLICT OF INTEREST

The authors declare no competing interests.

## AUTHORS CONTRIBUTION

Conception and design of research (Florian K. Paternoster, Wolfgang Seiberl), acquisition of data (Florian K. Paternoster, Denis Holzer, Anna Arlt), analysis and interpretation of data (Florian K. Paternoster, Denis Holzer, Anna Arlt, Ansgar Schwirtz, Wolfgang Seiberl), writing, and approval of the final version of the manuscript (Florian K. Paternoster, Denis Holzer, Anna Arlt, Ansgar Schwirtz, Wolfgang Seiberl).

## Supporting information



Fig S1

Table S1

Supplementary Material

## Data Availability

The datasets generated during and/or analyzed during the current study are available from the corresponding author on reasonable request.
