# Supplementary figures and images for: Residual force enhancement in humans: Is there a true non‐responder?
Source: Physiol Rep. 2021 Aug 2;9(15):e14944. doi: 10.14814/phy2.14944 (PMC8327164; doi:10.14814/phy2.14944)

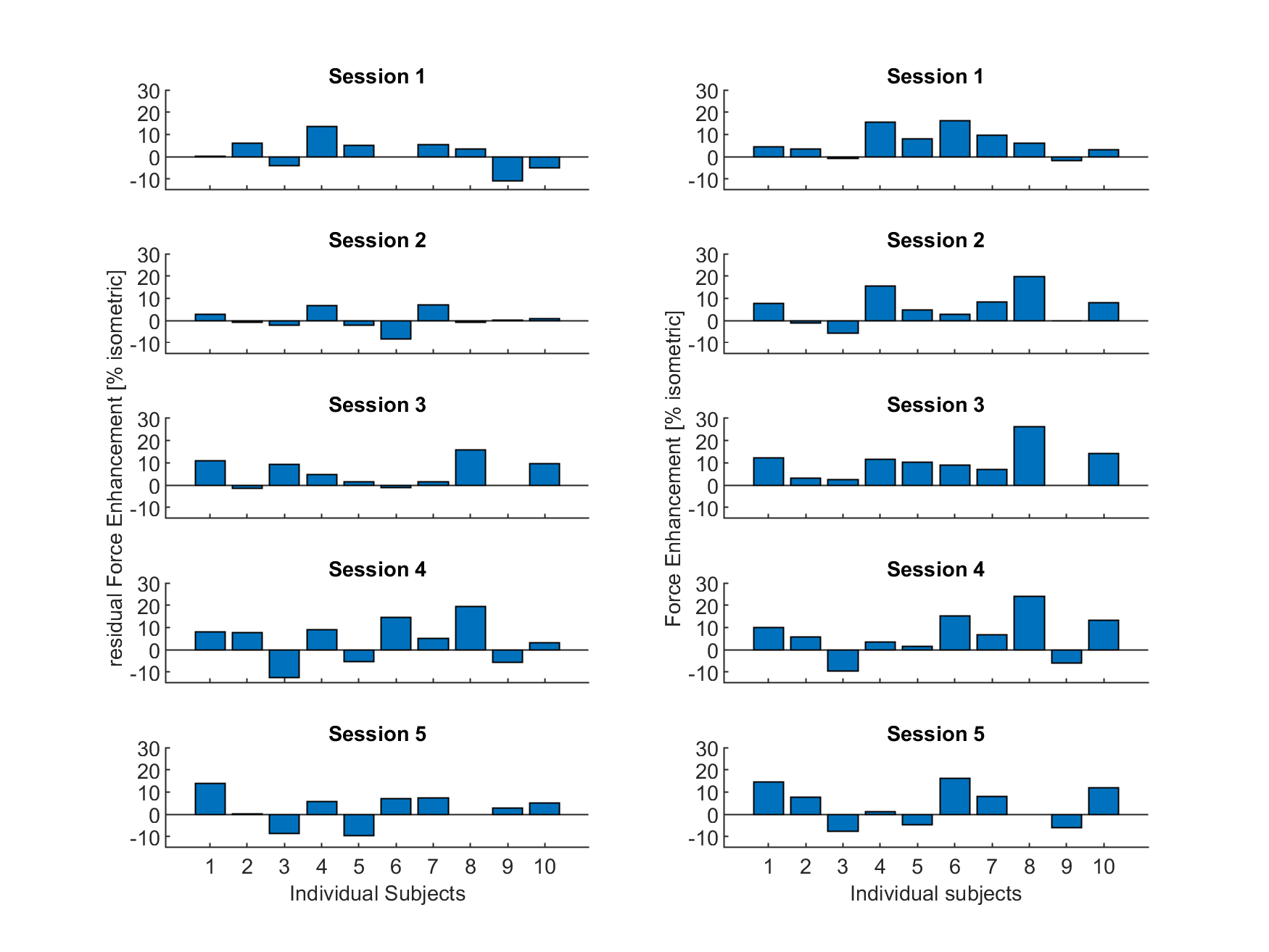

Supplement: Supplementary file 1 — Fig S1 [file PHY2-9-e14944-s003.tif]
