# Supplementary material for: Residual force enhancement in humans: Is there a true non‐responder?
Source: Physiol Rep. 2021 Aug 2;9(15):e14944. doi: 10.14814/phy2.14944 (PMC8327164; doi:10.14814/phy2.14944)
Supplement: Supplementary file 2 — Table S1 [file PHY2-9-e14944-s002.pdf]

The following analysis of triceps surae and tibialis anterior EMG data,
corresponds to the manuscript with the title:
Residual force enhancement: Is there a true non-responder?

EMG data is normalized using a task-specific maximum voluntary contraction

Data represents mean muscle activity between 2-2.5s after subjects
reached the individual torque treshold of 95% of maximum voluntary contraction

dynamic = isometric-eccentric-isometric contraction
reference = pure isometric contraction without preceeding stretch

	Gastrocnemius Lateralis		
	dynamic [% MVC]	reference [%MVC]	Statistics
Session 1	73.1 ± 9.6	76.3 ± 10.4	t(8) = -1.87, p = 0.098, d = -0.62
Session 2	59.8 ± 11.1	74.9 ± 15.2	t(8) = -2.58, p = 0.032, d = -0.86
Session 3	63.9 ± 15.1	71.5± 15.6	t(9) = -2.77, p = 0.022, d = -0.88
Session 4	74.0 ± 14.8	77.5 ± 15.7	t(9) = -0.55, p = 0.599, d = -0.17
Session 5	72.4 ± 17.7	74.3 ± 11.1	t(9) = -0.38, p = 0.716, d = -0.12

	Soleus		
	dynamic [% MVC]	reference [%MVC]	Statistics
Session 1	71.4 ± 7.7	76.6 ± 7.8	t(8) = -1.76, p = 0.116, d = -0.59
Session 2	67.9 ± 5.6	77.3 ± 5.4	t(8) = -3.44, p = 0.009, d = -1.15
Session 3	66.3 ± 9.5	72.6 ± 9.3	t(9) = -2.38, p = 0.041, d = -0.75
Session 4	69.7 ± 8.6	76.8 ± 11.2	t(8) = -1.81, p = 0.108, d = -0.60
Session 5	78.8 ± 16.0	81.4 ± 8.2	t(8) = -0.70, p = 0.506, d = -0.23

	Gastrocnemius Medialis		
	dynamic [% MVC]	reference [%MVC]	Statistics
Session 1	70 .2 ± 10.8	74.9 ± 6.2	t(7) = -2.61, p = 0.035, d = -0.92
Session 2	70.8 ± 11.4	75.2 ± 11.0	t(9) = -0.93, p = 0.378, d = -0.29
Session 3	67.6 ± 6.1	73.9± 9.2	t(8) = -1.33, p = 0.221, d = -0.44
Session 4	71.8 ± 10.6	76.5 ± 7.3	t(7) = -0.77, p = 0.468, d = -0.27
Session 5	74.3 ± 12.2	76.2 ± 6.8	t(8) = -0.27, p = 0.795, d = -0.09

	Tibialis anterior		
	dynamic [% MVC]	reference [%MVC]	Statistics
Session 1	73.6 ± 12.6	76.3 ± 10.4	t(8) = -1.96, p = 0.086, d = -0.65
Session 2	79.0 ± 10.4	74.9 ± 15.2	t(9) = -0.48, p = 0.643, d = -0.15
Session 3	79.4 ± 15.1	71.5± 15.6	t(8) = -2.76, p = 0.025, d = -0.92
Session 4	78.1 ± 10.0	77.5 ± 15.7	t(8) = 0.17, p = 0.871, d = 0.06
Session 5	81.1 ± 12.8	4.3 ± 11.1	t(9) = 0.15, p = 0.886, d = 0.05
