# Supplementary material for: Residual force enhancement in humans: Is there a true non‐responder?
Source: Physiol Rep. 2021 Aug 2;9(15):e14944. doi: 10.14814/phy2.14944 (PMC8327164; doi:10.14814/phy2.14944)
Supplement: Supplementary file 3 — Supplementary Material [file PHY2-9-e14944-s001.docx]

**Additional Files**

**S1 Table**

Detailed results and statistics of EMG analysis.

**S2 Figure**

Individual response of participants regarding residual force enhancement (left) and force enhancement (right). Note: Columns without a bar are treated as outliers (> mean ± 2* standard deviation).
